# Electronic Medical Record Data-Based Analysis of Discharge Pathways and Functional Recovery by Surgical Procedure Among Patients with Hip-Related Fractures in a Convalescent Rehabilitation Hospital: A Retrospective Cohort Study

**DOI:** 10.3390/medicina62061085

**Published:** 2026-06-03

**Authors:** Yong-Hwa Park, Bong-Sik Woo, Jung-Ho Lee

**Affiliations:** 1 IM Rehabilitation Hospital, 2140, Cheongnam-ro, Seowon-gu, Cheongju-si 28702, Chungcheongbuk-do, Republic of Korea; rmsid0245@naver.com (Y.-H.P.); wbongsky@daum.net (B.-S.W.); 2 Department of Physical Therapy, University of Kyungdong, 815, Gyeonhwon-ro, Munmak-eup, Wonju-si 26495, Gangwon-do, Republic of Korea

**Keywords:** hip, fracture, convalescent rehabilitation, discharge pathways, surgical procedure

## Abstract

*Background and Objectives*: Discharge planning after hip-related fracture surgery may depend on both the surgical method and functional recovery achieved during convalescent rehabilitation. This single-center retrospective cohort study aimed to determine whether discharge pathways differed according to surgical procedure and whether functional recovery patterns differed according to surgical procedure and discharge pathways among patients admitted for convalescent rehabilitation after hip-related fracture surgery. *Materials and Methods*: This retrospective cohort study reviewed the EMRs of patients admitted to a convalescent rehabilitation hospital between January 2021 and June 2025 after hip-related fracture surgery. Surgical groups were hip hemiarthroplasty (HA), total hip arthroplasty (THA), and internal fixation (IF). Discharge pathways were classified into three categories: home discharge, transfer to an acute-care hospital, and transfer to a long-term care hospital. In this study, home discharge was operationally defined as discharge to the patient’s home or transfer to a nursing hospital. Functional outcomes included the functional ambulation category (FAC), Berg Balance Scale (BBS), Modified Barthel Index (MBI), and Mini-Mental State Examination (MMSE); complete-case analysis was applied for functional outcomes. *Results*: In the overall postoperative cohort (N = 445), discharge pathway distributions differed across surgical groups. In the complete-case traumatic hip-related fracture cohort (N = 243), all groups showed significant improvements from admission to discharge in FAC, BBS, MBI, and MMSE. Between-group comparisons of change scores by surgical method were generally modest. In contrast, discharge pathways showed clearer associations with recovery. Patients achieving home discharge demonstrated greater improvements in FAC, BBS, and MBI measures than those transferred to acute care or nursing homes. *Conclusions*: Functional recovery was observed across all surgical groups during convalescent rehabilitation. Discharge disposition appeared to be more closely associated with recovery in gait, balance, and ADL performance than with surgical method alone; however, this finding should be interpreted cautiously because discharge decisions may also be influenced by patient and social factors.

## 1. Introduction

With rapid global population aging, the incidence of fall-related fractures is expected to rise substantially. Among older adults, hip-related fractures represent a major source of traumatic injury and are frequently associated with a cascade of adverse outcomes, including reduced mobility, prolonged hospitalization, and secondary complications. As a result, patients often experience functional decline and a reduced likelihood of community discharge, thereby increasing both societal and healthcare costs [1,2].

Early postoperative rehabilitation is a key component of recovery after hip-related fracture surgery. Evidence from hip fracture cohorts indicates that initiating rehabilitation as early as clinically feasible is associated with faster functional gains and lower risks of mortality and postoperative complications such as pneumonia, deep vein thrombosis, and pulmonary embolism [3,4]. These findings highlight the clinical importance of timely rehabilitation initiation and the establishment of an appropriate postoperative rehabilitation strategy, particularly for older patients who are vulnerable to immobility-related complications [3].

Convalescent rehabilitation hospitals provide an environment that supports intensive, multidisciplinary rehabilitation, and an adequate rehabilitation period can help ensure both the amount and quality of therapy while facilitating the recovery of independence in the activities of daily living [5,6]. Functional independence during convalescent rehabilitation is clinically important because it is closely linked to the feasibility of returning to the community after discharge. Prior studies have suggested that a higher intensity and frequency of therapeutic interventions, including physical therapy, is associated with favorable community discharge outcomes, and higher functional independence measure motor (FIM-motor) scores are consistently linked to higher rates of community discharge [7].

However, community discharge is not determined solely by physical function. Psychosocial and contextual factors such as cognitive function, depressive symptoms, cohabitation status, and the availability of social support have also been identified as important determinants of discharge to the community [8,9]. Therefore, discharge planning for patients with hip-related fractures requires a comprehensive approach that integrates functional recovery with psychological and social context.

In addition, surgical method may be associated with rehabilitation efficiency after hip-related fractures because it can influence early weight-bearing tolerance, pain, and the pace at which gait training can be safely implemented. In clinical practice, hip hemiarthroplasty (HA) and total hip arthroplasty (THA) are often selected to facilitate early mobilization in older adults, whereas internal fixation (IF) may require individualized postoperative weight-bearing restrictions depending on fracture stability and fixation quality [10]. In femoral neck fractures, surgical decision-making commonly considers displacement status (Garden classification) as well as patient-related factors such as age, bone quality, and premorbid activity level. Non-displaced fractures are often managed with internal fixation to preserve the native joint, whereas displaced fractures in older adults are frequently treated with HA or THA to facilitate earlier mobilization and reduce the risk of fixation failure or reoperation [11,12]. Thus, HA, THA, and IF represent clinically distinct pathways that may be associated with different functional recovery trajectories and discharge outcomes during convalescent rehabilitation [13].

Beyond clinical decision-making, institutional policy can also shape the organization and timing of convalescent rehabilitation. In the Republic of Korea, the convalescent rehabilitation medical institution system was introduced to provide intensive rehabilitation during the period of greatest functional recovery potential following acute care, with the aim of minimizing physical and cognitive impairments and facilitating patients’ early return to their homes and communities. Since its introduction, the system has been progressively refined based on operational data and clinical needs, including adjustments to admission criteria, recognized rehabilitation periods, and reimbursement structures to support patient-centered intensive rehabilitation [14].

In particular, policy revisions have attempted to reflect procedure-specific rehabilitation needs within the hip-related fracture category. A Ministry of Health and Welfare notification dated 28 February 2025 extended the allowable admission period from 30 to 60 days for selected procedures (internal fixation or total hip arthroplasty) within the hip-related fracture category, taking into account convalescent-phase rehabilitation characteristics such as postoperative muscle weakness and temporary weight-bearing restrictions. This revision can be interpreted as an institutional response to clinical realities in which gait retraining and balance and proprioceptive re-education may be delayed by early postoperative restrictions [15]. Therefore, the primary aim of this study was to clinically examine discharge pathways and functional recovery patterns after hip-related fracture surgery in a Korean convalescent rehabilitation setting, rather than to directly evaluate policy effects.

Notably, despite belonging to the broader category of arthroplasty, hip hemiarthroplasty was not included in the scope of this expanded application. Although HA typically involves shorter operative time and lower procedural complexity than THA, it is frequently performed in older patients with multiple comorbidities, and the delayed recovery of functional ambulation is not uncommon in clinical practice. This mismatch between policy assumptions and real-world rehabilitation trajectories raises two clinically and policy-relevant question: whether patients undergoing HA may require a rehabilitation period comparable to or longer than that of patients undergoing THA or IF, and whether surgical method is associated with differences in community discharge outcomes within convalescent rehabilitation settings [16].

Importantly, many patients with hip-related fractures undergo surgery at acute-care hospitals and are subsequently transferred to convalescent rehabilitation hospitals for intensive postoperative rehabilitation. Nevertheless, empirical evidence comparing discharge pathways and functional recovery patterns across surgical methods, specifically in the convalescent rehabilitation phase, remains limited. Addressing this gap may help inform procedure-sensitive rehabilitation planning and provide relevant evidence for the future refinement of admission criteria and reimbursement policies. Previous international studies have also suggested that discharge outcomes and functional recovery after hip surgery may vary according to patient profile, surgical indication, and postoperative rehabilitation context [4,8].

The present retrospective cohort study aimed to examine differences in community discharge rates and discharge pathways according to surgical methods among patients with hip-related fractures who were admitted to a convalescent rehabilitation medical institution after undergoing surgery at an acute-care hospital. We focused on three surgical methods and investigated whether procedure type was associated with differences in functional recovery patterns and community discharge outcomes during convalescent rehabilitation. Through these analyses, this study aims to provide evidence regarding procedure-associated differences in rehabilitation trajectories and discharge outcomes among patients with hip-related fractures in convalescent rehabilitation settings, and to inform future improvements in the operation of convalescent rehabilitation medical institutions and related reimbursement policies.

## 2. Materials and Methods

### 2.1. Study Design and Setting

This was a single-center retrospective cohort study based on consecutive eligible admissions identified from the electronic medical record system (Phoenix PLUS, Ginus, Inc., Seoul, Republic of Korea) during the study period. The study hospital was a convalescent rehabilitation institution in Korea; however, because this was a single-center study, the findings may not fully represent the characteristics of all convalescent rehabilitation hospitals nationwide. We screened admissions between January 2021 and June 2025 and retrospectively extracted clinical and rehabilitation assessment data from admission to discharge. The cohort entry (index date) was defined as the date of admission to the convalescent rehabilitation hospital following surgery performed at an external acute-care hospital. This study was conducted according to the Declaration of Helsinki and was approved by the Institutional Review Board (KNUT-2025-HR-16-34, Approval date: 8 August 2025).

### 2.2. Participants

**Figure 1:**
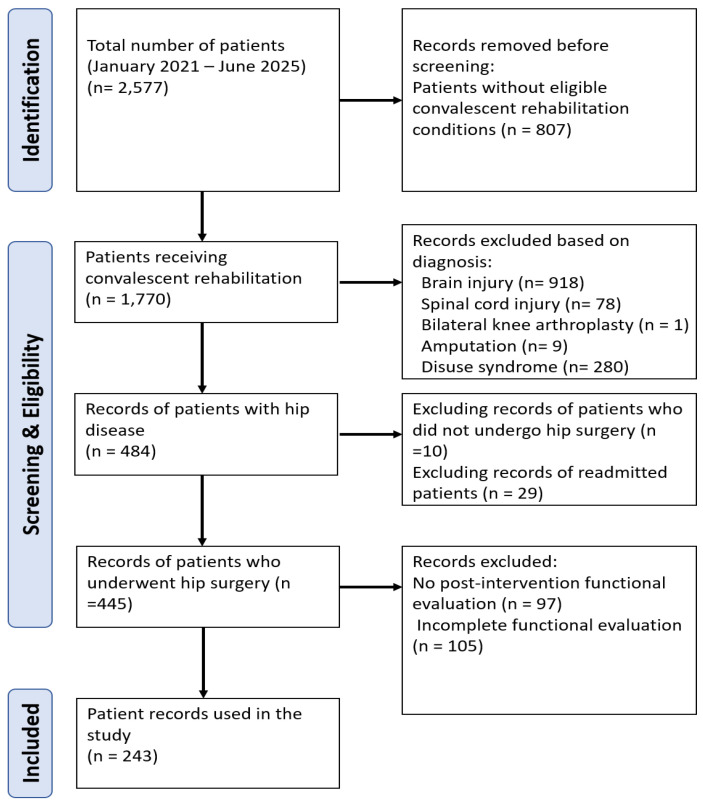
Identification of patients via databases and registers.

During the study period, 2577 admissions to the rehabilitation hospital were initially screened (Figure 1). Among these, 807 admissions that did not meet the criteria for convalescent rehabilitation were excluded, leaving 1770 admissions receiving convalescent rehabilitation therapy. These admissions were then screened by diagnostic category, and admissions with diagnoses other than hip-related conditions were excluded (brain injury, spinal cord injury, bilateral knee arthroplasty, amputation, and disuse syndrome), resulting in 484 admissions with hip-related conditions.

Among the 484 admissions with hip-related conditions, postoperative admissions were first identified for the overall discharge-pathway analysis. Patients who did not undergo surgery (n = 10) were excluded. To ensure the independence of observations, readmissions (n = 29) were excluded, and only the first eligible admission episode per patient was included, yielding the overall postoperative cohort (N = 445). For functional outcome analyses, this postoperative cohort was further restricted to patients with traumatic hip-related fractures who underwent HA, THA, or IF and had complete functional assessment data at both admission and discharge. Admissions for non-traumatic indications, including avascular necrosis, osteoarthritis, and dislocation-related arthroplasty, were excluded from the fracture-specific functional analytic cohort. Finally, patients were excluded if any of the functional measures (FAC, BBS, MBI, or MMSE) were missing at either admission or discharge, yielding a final complete-case cohort of 243 patients. This two-stage analytic structure was used to distinguish institution-level discharge pathway patterns from fracture-specific functional recovery analyses conducted in a more clinically comparable population.

### 2.3. Definitions and Eligibility Criteria

Hip-related fractures were defined as fractures of the pelvis and femur that constitute the hip joint. Patients were included if they: were admitted to the convalescent rehabilitation hospital for postoperative rehabilitation after surgery performed at an external acute-care hospital; had hip-related fractures as defined above; underwent one of the following surgical methods: hip hemiarthroplasty (HA), total hip arthroplasty (THA), or internal fixation (IF); received rehabilitation therapy during hospitalization; and had complete functional assessment data (FAC, BBS, MBI, and MMSE) recorded at both admission (baseline) and discharge.

Patients were excluded if they: received conservative treatment without surgery; underwent surgery for non-traumatic conditions (e.g., osteoarthritis or avascular necrosis); were readmitted during the study period (only the first eligible admission episode was retained); or had missing data for any of the required functional assessments (FAC, BBS, MBI, or MMSE) at admission or discharge.

### 2.4. Data Collection and Variable Definitions

Data were retrospectively collected from EMRs and included demographics (age, sex, height, weight), surgical method, discharge pathway, and functional outcome measures assessed at admission and discharge. Surgical method was categorized as hip hemiarthroplasty, total hip arthroplasty, or internal fixation. Accordingly, participants were classified into three groups (HA, THA, and IF). Discharge pathway was classified into three categories: home discharge, transfer to an acute-care hospital, and transfer to a long-term care hospital. In this study, ‘home discharge’ was operationally defined as discharge to the patient’s home or transfer to a nursing hospital within the Korean convalescent rehabilitation setting.

### 2.5. Outcome Measures

The FAC is an assessment tool used to evaluate the level of assistance required by a patient during ambulation. The FAC is scored on a scale from 0 to 5. A score of 0 indicates that ambulation is not possible or that assistance from two or more persons is required. A score of 1 indicates that continuous support from one person is necessary to maintain balance or to shift body weight. A score of 2 reflects the need for intermittent assistance from one person to support balance or coordination. A score of 3 indicates that ambulation is possible without physical contact, requiring only supervision or verbal cueing. A score of 4 denotes independent ambulation on level surfaces, with assistance required for stairs, ramps, or uneven surfaces. A score of 5 indicates fully independent ambulation [17].

The BBS is a widely used assessment tool designed to evaluate functional balance ability and fall risk. It consists of 14 functional tasks that range from static balance activities, such as sit-to-stand, sitting unsupported, and standing unsupported, to progressively more challenging dynamic balance tasks, including reaching forward, picking up an object from the floor, turning 360 degrees, stepping onto and off a stool, and standing on one leg. Each item is scored on a scale from 0 to 4, yielding a maximum total score of 56 points. Lower scores indicate poorer balance ability and a higher risk of falls. For a clinical interpretation of the functional levels, total BBS scores can be categorized as follows: 0–20 points indicate ambulation with assistive devices, 21–40 points indicate ambulation requiring assistance, and 41–56 points indicate independent ambulation. In terms of fall risk prediction, scores of 41–56 indicate low risk, 21–40 indicate moderate risk, and 0–20 indicate high fall risk [18].

The MBI is an assessment tool used to quantify functional independence by evaluating a patient’s performance in activities of daily living (ADL). It consists of 10 items, including personal hygiene, bathing, feeding, toileting, dressing, stair climbing, bowel control, bladder control, ambulation or wheelchair mobility, and transfers between a chair and a bed. For patients who are unable to ambulate, the wheelchair mobility item is applied instead of the ambulation item. Each item is rated on a five-level scale according to the level of assistance required, with scores assigned in a stepwise manner ranging from complete independence to total dependence. A total score of 100 indicates full independence across all items, and higher scores reflect better ADL performance. The MBI demonstrates a single-factor structure representing overall ADL function [19].

### 2.6. Rehabilitation Program

Convalescent rehabilitation therapy was provided to patients admitted after surgery for hip-related fractures with the primary goal of restoring ambulation and ADL through the improvement of joint range of motion, lower-extremity strength, balance, and functional mobility. Rehabilitation was delivered by a multidisciplinary team, primarily physical therapists and occupational therapists, and followed an institution-based framework that was progressively advanced according to each patient’s functional status and recovery stage.

**Figure 2:**
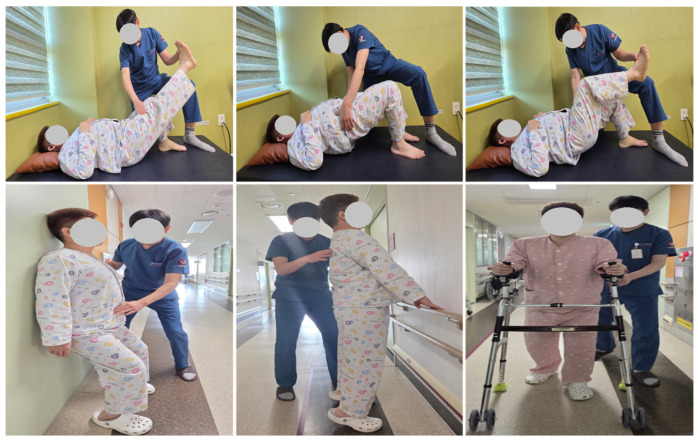
Convalescent physical therapy for patients undergoing hip surgery.

Physical therapy primarily consisted of range-of-motion exercise, progressive strengthening, balance training, and gait training, with an emphasis on gradually improving lower-extremity strength and weight-bearing tolerance while maintaining hip joint stability and ensuring patient safety (Figure 2). In the early phase, non-weight-bearing or low-load mat-based exercises were prioritized to minimize stress on the surgical site and facilitate basic muscle activation and strength recovery. As recovery progressed, therapy transitioned to partial weight-bearing and functional strengthening, followed by more advanced full weight-bearing tasks as tolerated. Examples of exercises used within this framework included ankle pumping, knee extension activation, straight leg raises, bridging, and progression to standing, or functional strengthening such as heel raises and wall squats, which were selected and modified according to individual functional levels and precautions.

**Figure 3:**
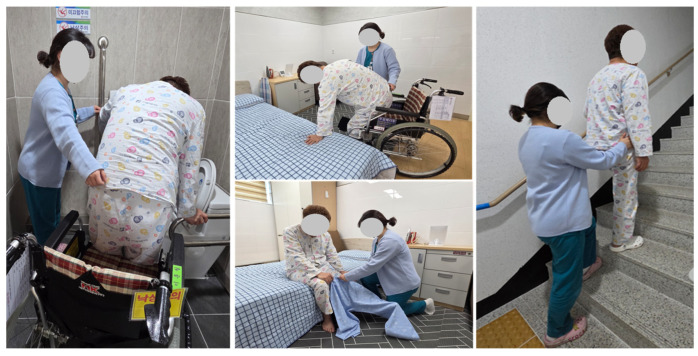
Convalescent occupational therapy for patients undergoing hip surgery.

Occupational therapy focused on restoring ADL performance and enhancing functional independence using task-oriented training closely aligned with real-life activities (Figure 3). Early stage training emphasized safe bed mobility and transfers with appropriate therapist assistance and assistive devices as needed. As functional capacity improved, training progressed to self-care tasks in simulated or real-life environments (dressing, toileting, grooming, and personal hygiene), incorporating adaptive strategies or assistive devices when indicated to accommodate hip precautions. In the later stage, OT expanded to ADL tasks integrated with gait and mobility tasks, including assisted device use, level-ground walking with directional changes, and a stepwise progression of walking speed and endurance. Stair negotiation and additional balance tasks were incorporated when clinically indicated to support safe mobility in home and community environments.

Rehabilitation intensity was prescribed using a unit-based framework (15 min/unit), with the daily dose adjusted by treating therapists according to each patient’s medical condition, weight-bearing status, pain, balance, and overall functional performance. However, because rehabilitation dose was individualized and not consistently retrievable in a standardized quantitative format from the EMR, detailed exposure measures such as total units per day, cumulative treatment time, or discipline-specific treatment dose could not be included in the analysis. Accordingly, variability in rehabilitation intensity should be regarded as a potential unmeasured confounder.

### 2.7. Statistical Analysis

Statistical analyses were performed using IBM SPSS Statistics version 22.0 (IBM Corp., Armonk, NY, USA). Descriptive statistics were used to summarize participant characteristics and key variables. Continuous variables are presented as mean ± standard deviation, and categorical variables are presented as a number (%). The normality of data distribution was assessed using the Shapiro–Wilk test. The homogeneity of variances was also assessed before parametric between-group comparisons. Analyses were conducted in two predefined analytic samples. First, discharge pathways were compared across surgical methods in the overall postoperative cohort (N = 445) to describe institutional discharge pathway distributions across procedures represented in the EMR dataset. Second, functional outcomes were analyzed in the traumatic hip-related fracture cohort with complete functional assessment data at both admission and discharge (complete-case cohort; N = 243), because change-score analyses required paired observations across time points. This analytic distinction was intended to separate descriptive discharge pathway patterns from clinically more comparable fracture-specific functional recovery analyses. A complete-case approach was selected because the primary functional analyses focused on within-patient change from admission to discharge across FAC, MMSE, BBS, and MBI. However, excluding patients with incomplete assessment data may have introduced selection bias if missingness was related to severity, transfer status, or rehabilitation participation.

In the complete-case cohort, baseline characteristics were compared across surgical method groups (HA vs. THA vs. IF). One-way ANOVA was used for continuous variables (e.g., age, height, weight, BBS, MBI, and MMSE), whereas nominal categorical variables, including sex (male/female), were compared using the chi-square (χ^2^) test. Within-group changes from admission to discharge were evaluated using paired *t*-tests for BBS, MBI, and MMSE. Between-group differences in change scores were examined using one-way ANOVA with Bonferroni-adjusted post hoc comparisons when applicable. As the FAC is an ordinal measure (coded 0–5), non-parametric methods were applied for FAC analyses. Within-group changes were assessed using the Wilcoxon signed-rank test, and between-group comparisons were conducted using the Kruskal–Wallis test with Bonferroni-adjusted pairwise comparisons when results were significant. All tests were two-tailed, and the level of statistical significance was set at α = 0.05.

## 3. Results

### 3.1. General Characteristics of the Overall Study Population

A total of 445 patients were included in the analysis, comprising 175 patients in the HA group, 108 patients in the THA group, and 162 patients in the IF group (Table 1). Of the total study population, 114 patients (25.6%) were male, and 331 patients (74.4%) were female. The mean age was highest in the HA group (84.19 ± 6.41 years), followed by the IF group (79.54 ± 14.75 years) and the THA group (73.22 ± 13.55 years). With respect to height, the THA group had the greatest mean height (159.86 ± 8.25 cm), whereas the HA and IF groups showed mean heights of 154.84 ± 13.07 cm and 157.72 ± 9.05 cm, respectively. Body weight was similarly distributed across the three groups, with mean values of 51.86 ± 8.94 kg in the HA group, 58.96 ± 9.34 kg in the THA group, and 58.39 ± 9.48 kg in the IF group.

Regarding the site of injury, single-site injuries accounted for the majority of cases in all three groups, with 156 patients (35.1%) in the HA group, 103 patients (23.1%) in the THA group, and 138 patients (31.2%) in the IF group. Fracture was the most common cause of injury, observed in 175 patients (39.3%) in the HA group, 43 patients (9.6%) in the THA group, and 162 patients (36.4%) in the IF group. Avascular necrosis (AVN) was observed only in the THA group (n = 36, 8.1%), and osteoarthritis was also identified exclusively in the THA group (n = 27, 6.2%). With respect to discharge pathways, discharge to the community was the most frequent outcome across all three groups, occurring in 123 patients (27.6%) in the HA group, 96 patients (21.5%) in the THA group, and 118 patients (26.5%) in the IF group. Transfer to an acute-care hospital occurred in 19 patients (4.2%) in both the HA and IF groups, whereas only 3 patients (0.8%) in the THA group were transferred to an acute-care hospital. Discharge to a long-term care hospital was observed in 33 patients (7.4%) in the HA group, 9 patients (2.2%) in the THA group, and 25 patients (5.6%) in the IF group.

### 3.2. Comparison of Discharge Pathways According to Surgical Method (Overall Population)

A chi-square test was conducted to compare the distribution of discharge pathways according to surgical method (Table 2). The results revealed a significant difference in discharge pathway distribution across surgical methods (χ^2^ = 29.240, *p* < 0.001). Residual analysis indicated that, in the THA group, the frequency of home discharge was significantly higher than the expected frequency (standardized residual = 2.4), whereas the frequencies of transfer to acute-care hospitals and long-term care hospitals were lower than expected (standardized residuals = −2.3 and −2.7, respectively).

In contrast, in the HA group, the frequency of transfer to long-term care hospitals was significantly higher than expected (standardized residual = 2.6), while home discharge showed a lower-than-expected tendency (standardized residual = −1.6). In the IF group, the frequency of discharge to acute-care hospitals was numerically higher than expected (standardized residual = 1.6); however, no marked differences were observed in discharge to the community or long-term care hospitals.

### 3.3. Baseline Characteristics and Functional Status of the Complete-Case Traumatic Hip-Related Fracture Cohort

At the initial postoperative assessment at admission, the mean age of the three groups was 84.05 ± 6.03 years in the HA group, 84.65 ± 7.32 years in the THA group, and 81.52 ± 11.15 years in the IF group (Table 3). The sex distribution consisted of 23 males and 96 females in the HA group, 6 males and 20 females in the THA group, and 21 males and 77 females in the IF group. With regard to physical characteristics, mean height was 154.17 ± 14.79 cm in the HA group, 158.46 ± 6.40 cm in the THA group, and 156.74 ± 8.70 cm in the IF group. Mean body weight was 52.60 ± 8.76 kg, 57.56 ± 9.85 kg, and 59.36 ± 9.42 kg in Groups 1, 2, and 3, respectively.

At the initial functional assessment, the median FAC score was 1.00 (0.00–1.00) in the HA group, 1.00 (0.00–1.75) in the THA group, and 1.00 (0.00–1.00) in the IF group. There was no significant difference in FAC among the three surgical groups at admission (Kruskal–Wallis H = 3.152, *p* = 0.207). MMSE scores were 16.81 ± 7.03, 18.34 ± 6.12, and 20.26 ± 6.81, respectively. BBS scores were 15.35 ± 11.90 in the HA group, 14.53 ± 11.36 in the THA group, and 14.39 ± 11.58 in the IF group, while MBI scores were 46.94 ± 18.43, 46.34 ± 18.59, and 48.01 ± 16.89 in the HA group, THA group, and IF group, respectively. As baseline MMSE differed significantly across surgical groups, Bonferroni-adjusted post hoc analysis was performed. The results showed that the IF group had significantly higher MMSE scores at admission than the HA group, whereas no significant differences were observed between the HA and THA groups or between the THA and IF groups. This baseline difference should be considered when interpreting rehabilitation participation, change scores, and discharge-related findings.

### 3.4. Within-Group Changes in Functional Outcomes from Admission to Discharge by Surgical Method (Complete-Case Cohort)

Within-group analyses comparing functional outcomes between admission and discharge demonstrated significant improvements across all surgical groups (Table 4). For BBS, MBI, and MMSE, paired *t*-tests showed significant increases from admission to discharge in each group (*p* < 0.001). For FAC, which was analyzed using the Wilcoxon signed-rank test, ambulatory independence increased significantly from admission to discharge in all groups (*p* < 0.001).

In the HA group, median FAC score increased from 1.00 (0.00–1.00) to 1.00 (1.00–2.00), MMSE from 16.81 ± 7.03 to 17.66 ± 6.99, BBS from 15.35 ± 11.90 to 20.92 ± 14.20, and MBI from 46.94 ± 18.43 to 52.81 ± 19.80. In the THA group, median FAC score increased from 1.00 (0.00–1.75) to 1.00 (1.00–2.00), MMSE from 18.34 ± 6.12 to 19.38 ± 6.74 (*p* = 0.003), BBS from 14.53 ± 11.36 to 22.30 ± 16.00, and MBI from 46.34 ± 18.59 to 52.61 ± 18.91. In the IF group, median FAC score increased from 1.00 (0.00–1.00) to 2.00 (1.00–2.00), MMSE from 20.26 ± 6.81 to 21.23 ± 7.23, BBS from 14.39 ± 11.35 to 21.88 ± 13.63, and MBI from 48.01 ± 16.89 to 55.54 ± 18.67 (*p* < 0.001).

### 3.5. Between-Group Comparisons of Functional Outcome Changes by Surgical Method (Complete-Case Cohort)

Between-group comparisons of change scores from admission to discharge showed no significant differences among surgical method groups for MMSE, BBS, or MBI (Table 5). Mean MMSE changes were 0.84 ± 2.09 in the hip hemiarthroplasty group, 1.03 ± 1.61 in the total hip arthroplasty group, and 0.96 ± 2.62 in the internal fixation group (one-way ANOVA, *p* = 0.891). Mean BBS changes were 5.57 ± 7.53, 7.76 ± 8.02, and 7.48 ± 9.50, respectively (*p* = 0.188), and mean MBI changes were 5.86 ± 8.56, 6.26 ± 7.41, and 7.85 ± 8.46, respectively (*p* = 0.304). Bonferroni-adjusted post hoc comparisons did not identify significant pairwise differences for these outcomes.

For FAC, the between-group comparison of change according to surgical method did not show a significant difference (Kruskal–Wallis H = 2.240, *p* = 0.326), suggesting that the magnitude of ambulatory improvement during hospitalization was not significantly associated with surgical procedure.

### 3.6. Between-Group Comparisons of Functional Outcomes by Discharge Pathway (Complete-Case Cohort)

Comparisons of change scores from admission to discharge according to discharge pathway are presented in Table 6. For BBS and MBI, one-way ANOVA revealed significant differences among discharge pathway groups (BBS: F = 3.624, *p* = 0.028; MBI: F = 3.513, *p* = 0.031) (Table 6). Bonferroni-adjusted post hoc comparisons indicated that the home discharge group showed greater improvements than the acute-care hospital and long-term care hospital groups for BBS, and greater improvements than the acute-care hospital transfer group for MBI. MMSE change did not differ significantly by discharge pathway (F = 1.152, *p* = 0.318).

For FAC, which was analyzed using the Kruskal–Wallis test, the magnitude of ambulatory improvement differed significantly according to discharge pathway (H = 25.509, *p* < 0.001). Bonferroni-corrected pairwise comparisons showed that the home discharge group demonstrated significantly greater FAC improvement than both the acute-care hospital transfer group and the long-term care hospital transfer group.

## 4. Discussion

The present retrospective cohort study examined discharge pathways and functional recovery patterns among patients admitted to a convalescent rehabilitation hospital after surgery for hip-related conditions, with a focus on differences according to surgical method and discharge pathway. The key findings can be summarized as follows. First, in the overall postoperative cohort, discharge pathway distributions differed by surgical group, with a relatively higher proportion of home discharge in the total hip arthroplasty group, whereas the hip hemiarthroplasty group showed a higher tendency toward transfer to nursing homes, and the internal fixation group more frequently required transfer to acute-care hospitals. Second, in the fracture-surgery complete-case cohort, functional outcomes improved significantly from admission to discharge across all surgical groups, evidencing the clinical value of convalescent rehabilitation during the early recovery period. Third, although between-group differences in the magnitude of improvement by surgical method were generally modest across outcomes, discharge pathway was more clearly associated with the magnitude of functional gains, particularly in gait-related function (FAC), balance (BBS), and activities of daily living (MBI). Collectively, these findings suggest that, although discharge pathway distributions differed across surgical groups in the overall postoperative cohort, the magnitude of functional improvement in the fracture-specific complete-case cohort was more clearly associated with the discharge pathway than with the surgical method itself. However, because this was a retrospective observational study, these findings indicate associations rather than causal relationships. In particular, discharge destination may be shaped not only by functional recovery during rehabilitation but also by patient characteristics and social or environmental factors that are not fully captured in the present dataset.

An interpretation of these findings is that convalescent rehabilitation functions as a recovery amplifier after hip-related surgery. While surgical method determines early postoperative constraints, the extent to which patients regain gait, balance, and ADL independence during convalescent hospitalization may exert a decisive influence on discharge planning. This interpretation is consistent with prior reports emphasizing that supervised and individualized rehabilitation promotes functional recovery after hip arthroplasty and that the early initiation of exercise therapy can improve gait and balance, reduce falls, and enhance quality of life even within short postoperative intervals [20,21]. In addition, early ambulation and structured exercise programs may shorten bed-rest duration and accelerate functional recovery [20]. Early postoperative rehabilitation generally focuses on restoring joint range of motion, strengthening peri-hip musculature, improving gait and balance, and recovering capacity for basic activities of daily living [21,22,23]. Despite differences in indication between total hip arthroplasty and hip hemiarthroplasty, postoperative rehabilitation remains an important factor influencing functional recovery across surgical approaches [24].

### 4.1. Discharge Pathway According to General Characteristics and Surgical Method (Overall Postoperative Cohort)

In the overall postoperative cohort (N = 445) admitted for convalescent rehabilitation between January 2021 and June 2025, patients were categorized into three surgical groups. The sex distribution showed the predominance of women, consistent with prior reports indicating that hip fractures occur approximately 2.5~3 times more frequently in women than in men [25]. This sex disparity has been linked to biological and functional vulnerabilities in older women including postmenopausal bone mineral density loss, reduced muscle strength and balance, and increased fall risk, suggesting that sex-related considerations may be relevant when designing rehabilitation strategies for older adults with hip-related fractures.

The age profile differed across surgical groups, with the HA group generally comprising older patients, whereas the THA group tended to include relatively younger and more physically active patients. This pattern is consistent with prior evidence that HA is often selected for older individuals or those with higher medical comorbidity due to shorter operative time and lower blood loss [26,27]. In contrast, the selection of THA is influenced by patients’ functional expectations, baseline health status, and anticipated postoperative mobility requirements [28].

Differences in discharge pathway distribution across surgical groups were observed, with a relatively higher proportion of home discharge (home or long-term care hospital) in the THA group. This finding may reflect a combination of factors such as younger age, fewer weight-bearing restrictions, lower complication burden, and higher baseline functional reserve. In contrast, the HA group showed a greater tendency toward transfer to nursing homes, while the IF group more frequently required transfer to acute-care hospitals, suggesting that age, functional status, complication risk, and postoperative restrictions may collectively shape discharge pathways [29,30].

### 4.2. Baseline Characteristics and Initial Functional Status in the Fracture-Surgery Cohort (Complete-Case Cohort)

In the fracture-surgery cohort, analyzed using complete functional assessment data, baseline characteristics and admission functional measures were generally comparable across surgical groups, supporting the interpretability of subsequent comparisons in functional recovery trajectories. This approach aligns with prior studies emphasizing the importance of accounting for baseline functional status when evaluating prognoses after hip fracture rehabilitation [31]. The lack of substantial baseline differences in admission functional measures may reflect the immediate postoperative context in which pain, restricted weight bearing, and early immobilization can attenuate observable intergroup differences; such differences may become more apparent later in recovery [28,30].

However, differences in admission MMSE were observed, with relatively higher scores in the internal fixation group. This suggests that preserved cognitive function may facilitate greater rehabilitation participation and more effective task learning during convalescent rehabilitation, and is consistent with prior evidence that cognitive status predicts functional recovery and discharge outcomes after hip fracture [29]. As baseline MMSE differed significantly across surgical groups, this difference should be considered when interpreting subsequent rehabilitation participation, recovery patterns, and discharge outcomes.

### 4.3. Functional Improvements from Admission to Discharge According to Surgical Method

Across all surgical groups, functional outcomes improved from admission to discharge, including gait-related function, cognitive function, balance, and ADL. These findings support the clinical value of convalescent rehabilitation in promoting recovery after surgery in patients with hip-related fractures, consistent with reports that intensive rehabilitation during the convalescent phase contributes to improvements in functional independence [32].

When comparing the magnitude of change across surgical methods, differences were generally modest across outcomes. In particular, while gait-related improvement appeared greater in the IF group compared with the HA group, this pattern should be interpreted cautiously as a tendency rather than definitive superiority unless it is supported by a statistically significant overall group comparison and appropriately adjusted post hoc testing. Overall, these findings suggest that the surgical method alone may not be the primary determinant of convalescent functional gains; instead, factors such as baseline reserve, allowable weight bearing, complication burden, pain control, and rehabilitation participation may play more direct roles in shaping recovery trajectories [33,34]. Taken together, these findings indicate that the magnitude of short-term functional improvement did not differ significantly according to surgical method in the fracture-specific complete-case cohort.

### 4.4. Association Between Functional Improvement and Discharge Pathway

In the fracture-surgery cohort, between-group comparisons indicated that most functional change scores did not differ substantially by surgical method. This pattern supports the interpretation that convalescent rehabilitation yields meaningful gains across surgical approaches and that recovery magnitude is influenced by multifactorial clinical and environmental determinants beyond surgical type alone, including baseline functional status, weight-bearing allowance, pain and complication management, and treatment engagement [29,34]. Ambulatory improvement assessed using the FAC showed significant within-group improvement across all surgical groups, whereas between-group differences by surgical method were not significant. By contrast, FAC improvement differed significantly according to discharge pathway, with greater improvements observed in the home discharge group.

In contrast to surgical method, discharge pathway was more clearly associated with functional recovery patterns. Gait-related improvement (FAC) differed across discharge pathways, with the home discharge group demonstrating greater improvements than the groups transferred to acute-care hospitals or long-term care hospitals. This finding suggests that the gait independence achieved during convalescent rehabilitation is a key functional determinant of discharge disposition. Balance improvement (BBS) also differed by discharge pathway, with greater gains in the home discharge group compared with the long-term care hospital transfer group. As balance is directly related to fall risk and safe community mobility, these results indicate that postural control may be an important factor in discharge decision-making beyond ambulatory capacity alone, consistent with prior reports linking impaired balance to institutional discharge after fractures [21,35]. At the same time, discharge pathway should be interpreted not only as an outcome of inpatient recovery but also as a potential marker of unmeasured factors such as caregiver availability, premorbid living circumstances, and social support.

Similarly, ADL improvement (MBI) differed by discharge pathway, with greater gains in the home discharge group than in the acute-care hospital transfer group. This pattern suggests that discharge to home settings reflects not only medical stability but also the attainment of functional independence which is sufficient for basic self-care and mobility demands; this is consistent with evidence linking functional independence to post-discharge living arrangements [29]. MMSE-change did not differ significantly by discharge pathway. This may reflect the possibility that discharge decisions during convalescent rehabilitation are driven more directly by physical functional status than by short-term cognitive change. Nonetheless, cognitive function has been implicated in rehabilitation participation and longer-term community reintegration, and its impact may be more evident in longitudinal follow-up beyond discharge [36]. Accordingly, the observed relationship between greater functional improvement and home discharge should be interpreted as an association within the convalescent rehabilitation context, rather than evidence that functional gain alone determines discharge disposition.

### 4.5. Policy Implication and System-Level Interpretation

The convalescent rehabilitation medical institution system in the Republic of Korea was introduced to provide intensive rehabilitation during the period of greatest recovery potential after acute care, with the goal of improving functional independence and facilitating a return to home and community settings [9]. A revision issued on 28 February 2025 expanded the recognized treatment window from 30 to 60 days for selected postoperative groups, including patients with hip, pelvic, or femoral fractures or arthroplasty who underwent internal fixation or total hip arthroplasty [14,15]. However, hip hemiarthroplasty remained outside this expansion.

In the present study, the hip hemiarthroplasty group showed a higher frequency of transfer to a nursing home compared with the other surgical groups. This pattern may be interpreted as a reflection of an institutional gap. Patients undergoing hip hemiarthroplasty are often older and medically vulnerable, and may exhibit slower recovery trajectories, yet may be allocated a shorter recognized intensive rehabilitation period. If convalescent rehabilitation is terminated before sufficient functional gains are achieved, the probability of institutional transfer may increase. These findings support the consideration that the recognized rehabilitation period for hip hemiarthroplasty should be extended to better align it with real-world recovery trajectories, and future policy refinements guided by empirical evidence on functional improvement patterns and discharge outcomes.

### 4.6. Limitations and Future Research Directions

This study has limitations. Rehabilitation dose was individualized according to patient status and could not be quantified reliably from EMRs, which may introduce residual confounders related to unmeasured therapy intensity. In addition, we did not perform multivariable regression adjustments for age, baseline cognition, comorbidity burden, or other potential confounders. Therefore, the present findings should be interpreted as unadjusted associations rather than independent procedure-specific effects. In addition, because functional analyses were based on complete cases only, selection bias cannot be excluded if patients with missing admission or discharge assessments differed systematically in terms of medical stability, rehabilitation tolerance, or discharge trajectory. Discharge pathway decisions may reflect both functional status and non-medical factors such as caregiver availability and community resources, which were not fully captured in the current dataset. In addition, because discharge terminology may differ across healthcare systems, the category of home discharge in this study should be understood as a study-specific operational term indicating discharge to the patient’s home or transfer to a nursing hospital within the Korean convalescent rehabilitation context.

Future studies should validate these findings in multicenter cohorts to enhance generalizability across different convalescent rehabilitation settings and regional practice patterns. Prospective designs are particularly needed to quantify rehabilitation dose (therapy minutes, intensity, content, and progression) and to clarify dose–response relationships with functional recovery and discharge disposition. In addition, because discharge decisions are influenced by both clinical and non-clinical factors, future work should incorporate detailed social determinants and contextual variables, including caregiver availability, living environment, socioeconomic status, and access to community resources, to better explain discharge pathways beyond functional measures alone.

## 5. Conclusions

In this retrospective cohort study of patients admitted to a convalescent rehabilitation hospital after hip-related fracture surgery, discharge pathways differed by surgical method in the overall postoperative cohort. In the fracture-specific complete-case cohort, functional outcomes improved from admission to discharge across surgical groups, whereas between-group differences in the magnitude of change by surgical method were not statistically significant. By contrast, functional improvement during convalescent rehabilitation was more clearly associated with discharge pathway. These findings suggest that recovery in gait, balance, and ADL performance may be closely related to discharge disposition, and highlight the potential importance of individualized rehabilitation strategies, although the results should be interpreted cautiously given the retrospective design and potential confounders.

## Figures and Tables

**Table 1:** General characteristics and discharge destinations by surgical methods (N = 445).

		HA (n = 175)	THA (n = 108)	IF (n = 162)
Age (year)		84.19 (6.41)	73.22 (13.55)	79.54 (14.75)
Sex (M/F)		32/143	39/69	43/119
Height (cm)		154.84 (13.07)	159.86 (8.25)	157.72 (9.05)
Weight (kg)		51.86 (8.94)	58.96 (9.34)	58.39 (9.48)
Injury site	Multiple sites (n/%)	19 (4.2)	5 (1.1)	24 (5.3)
	Single site (n/%)	156 (35.1)	103 (23.14)	138 (31.2)
Etiology	AVN (n/%)	0	36 (8.1)	0
	Osteoarthritis (n/%)	0	27 (6.2)	0
	Fracture (n/%)	175 (39.3)	43 (9.6)	162 (36.4)
	Dislocation (n/%)	0	2 (0.4)	0
Discharge pathway	Home discharge (n/%)	123 (27.6)	96 (21.5)	118 (26.5)
	Acute-care hospital (n/%)	19 (4.2)	3 (0.8)	19 (4.2)
	Long-term care hospital (n/%)	33 (7.4)	9 (2.2)	25 (5.6)

HA: hip hemiarthroplasty; THA: total hip arthroplasty; IF: internal fixation; AVN: avascular necrosis. Values are presented as mean ± standard deviation or number/%.

**Table 2:** Discharge pathways by surgical method in the overall postoperative cohort (N = 445).

Discharge Pathway		HA (n = 175)	THA (n = 108)	IF (n = 162)
Home discharge	Observed frequency (n/%)	123 (70.3%)	96 (88.9%)	118 (72.8%)
	Expected frequency	139.0	85.5	122.5
	Standardized residual	−1.6	2.4	−0.4
Acute-care hospital	Observed frequency (n/%)	19 (10.8%)	3 (2.8%)	19 (11.7%)
	Expected frequency	17.9	9.9	13.2
	Standardized residual	0.3	−2.3	1.6
Long-term care hospital	Observed frequency (n/%)	33 (18.9%)	9 (8.3%)	25 (15.5%)
	Expected frequency	19.1	21.7	27.3
	Standardized residual	2.6	−2.7	−0.4

HA: hip hemiarthroplasty; THA: total hip arthroplasty; IF: internal fixation.

**Table 3:** Baseline characteristics and functional status at admission among patients with traumatic hip-related fractures by surgical method (complete-case cohort, N = 243).

	HA (n = 119)	THA (n = 26)	IF (n = 98)	Test Statistic	*p*
Age (year)	84.05 (6.03)	84.65 (7.32)	81.52 (11.15)	2.824	0.061
Sex (M/F)	23/96	6/20	21/77	0.880	0.881
Height (cm)	154.17 (14.79)	158.46 (6.40)	156.74 (8.70)	2.038	0.132
Weight (kg)	52.60 (8.76)	57.56 (9.85)	59.36 (9.42)	1.043	0.354
FAC (score)	1.00 (0.00–1.00)	1.00 (0.00–1.75)	1.00 (0.00–1.00)	3.152	0.207
MMSE (score)	16.81 (7.03)	18.34 (6.12)	20.26 (6.81)	6.811	0.001 *
BBS (score)	15.35 (11.90)	14.53 (11.36)	14.39 (11.58)	0.194	0.824
MBI (score)	46.94 (18.43)	46.34 (18.59)	48.01 (16.89)	0.138	0.871

HA: hip hemiarthroplasty; THA: total hip arthroplasty; IF: internal fixation; FAC: functional ambulation category; MMSE: Mini-Mental State Examination; BBS: Berg Balance Scale; MBI: Modified Barthel Index. Values are presented as mean (SD), number, or median (Q1–Q3), as appropriate. Continuous variables (age, height, weight, MMSE, BBS, MBI) were compared using one-way ANOVA (F statistic); sex was compared using the chi-square test (χ^2^); FAC was compared using the Kruskal–Wallis test (H statistic). *: *p* < 0.05.

**Table 4:** Within-group changes in functional outcomes from admission to discharge by surgical method (complete-case cohort, N = 243).

Group	Variable	Pre-Test	Post-Test	t or Z	*p*
HA (n = 119)	FAC (score)	1.00 (0.00–1.00)	1.00 (1.00–2.00)	−7.065	<0.001 *
	MMSE (score)	16.81 (7.03)	17.66 (6.99)	−4.414	<0.001 *
	BBS (score)	15.35 (11.90)	20.92 (14.20)	−8.071	<0.001 *
	MBI (score)	46.94 (18.43)	52.81 (19.80)	−7.467	<0.001 *
THA (n = 26)	FAC (score)	1.00 (0.00–1.75)	1.00 (1.00–2.00)	−3.358	0.001 *
	MMSE (score)	18.34 (6.12)	19.38 (6.74)	−3.285	0.003 *
	BBS (score)	14.53 (11.36)	22.30 (16.00)	−4.939	<0.001 *
	MBI (score)	46.34 (18.59)	52.61 (18.91)	−4.310	<0.001 *
IF (n = 98)	FAC (score)	1.00 (0.00–1.00)	2.00 (1.00–2.00)	−6.603	<0.001 *
	MMSE (score)	20.26 (6.81)	21.23 (7.23)	−3.649	<0.001 *
	BBS (score)	14.39 (11.35)	21.88 (13.63)	−7.802	<0.001 *
	MBI (score)	48.01 (16.89)	55.54 (18.67)	−8.809	<0.001 *

HA: hip hemiarthroplasty; THA: total hip arthroplasty; IF: internal fixation; FAC: functional ambulation category; MMSE: Mini-Mental State Examination; BBS: Berg Balance Scale; MBI: Modified Barthel Index. Values are presented as mean (SD) or median (Q1–Q3). FAC was analyzed using the Wilcoxon signed-rank test (Z statistic), whereas BBS, MBI, and MMSE were analyzed using paired *t*-tests (t statistic). *: *p* < 0.05.

**Table 5:** Between-group comparisons of changes in functional outcomes by surgical method (complete-case cohort, N = 243).

Outcome	Surgical Method	Post-Pre Test	F or H	*p*
FAC (score)	HA (n = 119)	0.00 (0.00–1.00)	2.240	0.326
	THA (n = 26)	0.50 (0.00–1.00)		
	IF (n = 98)	1.00 (0.00–1.00)		
MMSE (score)	HA (n = 119)	0.84 (2.09)	0.115	0.891
	THA (n = 26)	1.03 (1.61)		
	IF (n = 98)	0.96 (2.62)		
BBS (score)	HA (n = 119)	5.57 (7.53)	1.681	0.188
	THA (n = 26)	7.76 (8.02)		
	IF (n = 98)	7.48 (9.50)		
MBI (score)	HA (n = 119)	5.86 (8.56)	1.073	0.304
	THA (n = 26)	6.26 (7.41)		
	IF (n = 98)	7.85 (8.46)		

HA: hip hemiarthroplasty; THA: total hip arthroplasty; IF: internal fixation; FAC: functional ambulation category; MMSE: Mini-Mental State Examination; BBS: Berg Balance Scale; MBI: Modified Barthel Index. Values are presented as mean ± standard deviation. For MMSE, BBS, and MBI, and as median (Q1–Q3) for FAC. For MMSE, BBS, and MBI, one-way ANOVA with Bonferroni-adjusted post hoc comparisons were used. FAC change was analyzed using the Kruskal–Wallis test.

**Table 6:** Between-group comparisons of changes in functional outcomes by discharge pathway (complete-case cohort, N = 243).

Outcome	Discharge Pathway	Post-Pre Test	F or H	*p*	Post Hoc
FAC (score)	Home discharge (n = 174)	1.00 (0.00–1.00)	25.509	<0.001 *	1 > 2, 3
	Acute-care hospital (n = 24)	0.00 (0.00–0.00)			
	Long-term care hospital (n = 45)	0.00 (0.00–1.00)			
MMSE (score)	Home discharge (n = 174)	1.00 (2.37)	1.152	0.318	
	Acute-care hospital (n = 24)	0.25 (2.02)			
	Long-term care hospital (n = 45)	0.95 (1.97)			
BBS (score)	Home discharge (n = 174)	7.48 (8.77)	3.624	0.028 *	1 > 2, 3
	Acute-care hospital (n = 24)	4.08 (5.97)			
	Long-term care hospital (n = 45)	4.40 (7.77)			
MBI (score)	Home discharge (n = 174)	7.37 (7.66)	3.513	0.031 *	1 > 2
	Acute-care hospital (n = 24)	2.91 (3.46)			
	Long-term care hospital (n = 45)	5.46 (8.88)			

FAC: functional ambulation category; MMSE: Mini-Mental State Examination; BBS: Berg Balance Scale; MBI: Modified Barthel Index. Values are presented as mean ± standard deviation. For MMSE, BBS, and MBI, and as median (Q1–Q3) for FAC. For MMSE, BBS, and MBI, one-way ANOVA with Bonferroni-adjusted post hoc comparisons were used. FAC change was analyzed using the Kruskal–Wallis test. *: *p* < 0.05.

## Data Availability

The original contributions presented in this study are included in the article. Further inquiries can be directed to the corresponding author.
